# Genome-wide identification and characterization of TCP gene family in *Dendrobium nobile* and their role in perianth development

**DOI:** 10.3389/fpls.2024.1352119

**Published:** 2024-02-05

**Authors:** Xinrui Wei, Meng Yuan, Bao-Qiang Zheng, Lin Zhou, Yan Wang

**Affiliations:** State Key Laboratory of Tree Genetics and Breeding; Research Institute of Forestry, Chinese Academy of Forestry, Beijing, China

**Keywords:** TCP gene family, expression pattern, perianth development, protein interaction, *Dendrobium nobile*

## Abstract

TCP is a widely distributed, essential plant transcription factor that regulates plant growth and development. An in-depth study of *TCP* genes in *Dendrobium nobile*, a crucial parent in genetic breeding and an excellent model material to explore perianth development in *Dendrobium*, has not been conducted. We identified 23 *DnTCP* genes unevenly distributed across 19 chromosomes and classified them as Class I PCF (12 members), Class II: CIN (10 members), and CYC/TB1 (1 member) based on the conserved domain and phylogenetic analysis. Most DnTCPs in the same subclade had similar gene and motif structures. Segmental duplication was the predominant duplication event for *TCP* genes, and no tandem duplication was observed. Seven genes in the CIN subclade had potential miR319 and -159 target sites. Cis-acting element analysis showed that most *DnTCP* genes contained many developmental stress-, light-, and phytohormone-responsive elements in their promoter regions. Distinct expression patterns were observed among the 23 *DnTCP* genes, suggesting that these genes have diverse regulatory roles at different stages of perianth development or in different organs. For instance, *DnTCP4* and *DnTCP18* play a role in early perianth development, and *DnTCP5* and *DnTCP10* are significantly expressed during late perianth development. *DnTCP17*, *20*, *21*, and *22* are the most likely to be involved in perianth and leaf development. *DnTCP11* was significantly expressed in the gynandrium. Specially, MADS-specific binding sites were present in most *DnTCP* genes putative promoters, and two Class I DnTCPs were in the nucleus and interacted with each other or with the MADS-box. The interactions between TCP and the MADS-box have been described for the first time in orchids, which broadens our understanding of the regulatory network of TCP involved in perianth development in orchids.

## 1 Introduction

TEOSINTE BRANCHED1, CYCLOIDEA, and PROLIFERATING CELL FACTORS (TCP) is a widely distributed, essential plant transcription factor that known after its first identified members: TEOSINTE BRANCHED1 (TB1) from *Zea mays*, CYCLOIDEA (CYC) from *Antirrhinum*, and PROLIFERATING CELL FACTORS (PCF) from *Oryza sativa* (Doebley et al., 1995; Luo et al., 1996; Kosugi and Ohashi, 1997). TCP has a highly conserved domain of approximately 59 amino acids, which has distinct characteristics, including nuclear targeting, DNA binding, and protein-protein interactions (Martín-Trillo and Cubas, 2010). TCP can be divided into two main classes: Class I (PCF or TCP-P) and Class II (TCP-C). The difference between these classes is embodied by the absence of four amino acids in the TCP basic region of Class I (Cubas et al., 1999). Moreover, Class II can be divided into two subclades: CINCINNATA (CIN) and CYC/TB1 (Navaud et al., 2007). Some members in the CYC/TB1 clade are found to contain an R-domain of unknown function, which is rarely in the CIN clade (Martín-Trillo and Cubas, 2010; Zhang et al., 2020).

CIN-TCP have been shown to influence plant morphological development by regulating the size or color of petals and leaves and creating wavy phenotypes (Uberti Manassero et al., 2013; Koyama et al., 2017; Zheng et al., 2022). Chimeric repressor silencing technology (CRES-T) applies this phenomenon to produce novel floral traits in horticultural plants of high ornamental value (Narumi et al., 2011; Ono et al., 2012; Zhang et al., 2021a). Meanwhile, CYC/TB1 members play crucial roles in lateral branching (Doebley et al., 1997; Aguilar-Martínez et al., 2007) and flower symmetry (Preston et al., 2011; Zhao et al., 2018; Pan et al., 2022). Unlike the functions of Class II members, most Class I TCP members remain unclear and have mainly been characterized in model plants (Viola et al., 2023). Class I *TCP* genes influence the development of leaves (Aguilar-Martínez and Sinha, 2013), cotyledons (Alem et al., 2022), stamens, gynoecium (Lucero et al., 2015), and seeds (Resentini et al., 2015) mainly through promoting or repressing cell proliferation and cell cycle-related processes. Previous studies have demonstrated that Class II *TCP* genes are involved in perianth development (Zhao et al., 2018; Pabón-Mora et al., 2020). However, the function of Class I *TCP* genes in perianth development remains unclear.

Given that the role of a single transcription factor is limited, TCP transcription factors (TCPs) play different roles in growth and development through interactions with other transcription regulators, which has received attention (Lucero et al., 2017; Zhao et al., 2023). Notably, MADS-box transcription factors involved in floral organ specification and perianth diversification have also been associated with TCPs (Dornelas et al., 2011; Shan et al., 2019; Ding et al., 2020). In *Arabidopsis thaliana*, *TCP* genes have been recognized as targets of SEPALLATA3 (SEP3) (Kaufmann et al., 2009), and AtTCP7, AtTCP8, and AtTCP15 were observed to directly activate the transcription of *SUPPRESSOR OF OVEREXPRESSION OF CONSTANS 1* (*SOC1*) to regulate flowering (Li et al., 2021; Camoirano et al., 2023). In *Gerbera hybrida*, GRCD5 (E-class MADS-box) contributes to ligule development by activating the expression of *GhCYC3* (Zhao et al., 2020). Additionally, CYC2-like proteins and ABCE-class MADS-box proteins interact to form complexes that determine perianth identity in *Chrysanthemum* (Wen et al., 2019).

Orchidaceae is one of the largest families of flowering plants (Cozzolino and Widmer, 2005). Most orchid species exhibit extreme diversity and specialization in perianth morphology, which is closely related to MADS-box transcription factors, as described in the “orchid code” proposed by Aceto and Gaudio (2011) and the “P code” proposed by Hsu et al. (2015). In *Phalaenopsis equestris*, CIN-TCPs could act as dual-function transcription factors by regulating petal and leaf size (Lin et al., 2016). In *Dendrobium chrysotoxum, DchCIN* genes could be involved in sepal and petal development, and DchTCP4 (Class I TCP) could significantly impact the phenotype of floral organs (Huang et al., 2023). There are few studies on the role of TCP in the flower development of orchids, let alone the involvement of TCP-MADS in flower development in orchids. Therefore, comprehending the molecular interactions between TCP and MADS-box is necessary to perform their role and is critical for understanding their system of action in regulating orchid flower development.

*Dendrobium nobile*, a zygomorphic (bilaterally symmetrical) flower with a highly specialized perianth like most plants in Orchidaceae, is a valuable genetic resource for flower improvement (Teixeira da Silva et al., 2014; Qiu et al., 2023). Recently, TCPs have been identified various plant species and shed information on their role in flower development. For instance, *Petunia axillaris*, *Catharanthus roseus*, *Cymbidium goeringii*, and *Chrysanthemum lavandulifolium* have 32, 15, 14, and 39 TCPs, respectively (Zhang et al., 2020; Liu D. K. et al., 2022; Hao et al., 2023; Wu et al., 2023). However, the role of TCP in flower development of *D. nobile* remain unclear. In this study, we aimed to identify putative *TCP* genes in the genome of *D. nobile* and their roles in flower development. To achieve this objective, we comprehensively analyzed the fundamental characteristics, phylogenetic relationships, miRNA target sites, codon usage biases, cis-acting elements, Gene ontology (GO) annotations, and protein-protein interactions of these TCPs. Notably, this study is the first to analyze the spatio-temporal expression patterns of *TCP* genes at four different perianth developmental stages (S1: unpigmented perianth; S2: initiate-pigmented perianth; S3: total-pigmented perianth; and S4: fully open perianth) in *D. nobile*. This analysis is substantially vital for orchid resource breeding and development. Moreover, this study is the first to describe the interaction between TCP and the MADS-box in orchids, which is a novel finding that broadens our understanding of the regulatory network of TCP involved in perianth development in orchids.

## 2 Materials and methods

### 2.1 Plant materials

The *D. nobile* plants were cultivated in the Chinese Academic Forestry greenhouse (Beijing, China) under natural light conditions, with daytime and night-time temperatures maintained at 25 ± 2°C. The study utilized four different perianth developmental stages: S1 (unpigmented perianth), S2 (initiate-pigmented perianth), S3 (total-pigmented perianth), and S4 (fully open perianth). In addition, the different organs, including the gynandrium, pedicel, and leaves, are at the stage of fully open flowers (S4). All plant materials were collected and stored at -80°C.

### 2.2 Data acquisition

The TCP protein sequences of *A. thaliana* and *O. sativ*a were obtained from TAIR (https://www.*Arabidopsis*.org/) and the *O. sativa* genome database (http://rice.plantbiology.msu.edu/), respectively (Martín-Trillo and Cubas, 2010). The *D. nobile* genome sequences were available for download from their whole-genome sequencing data (BioProject ID: PRJNA725550) (Xu et al., 2022). Furthermore, we also obtained TCP protein sequences from other orchid species, including *P. equestris* (Cai et al., 2015), *Dendrobium catenatum* (Zhang et al., 2021b), and *C. goeringii* (Liu D. K. et al., 2022). **Supplementary Table 1** displays each of the sequences above.

### 2.3 Identification of TCP gene family members from *D. nobile*

Firstly, the initial candidate members of the TCP gene family in *D. nobile* were identified by conducting a local BLAST search using 24 known AtTCPs and 22 OsTCPs from *A. thaliana* and *O. sativa* protein sequences as queries. Protein sequences with more than 50% similarity and e-values less than 1e-5 were selected. Then, the hidden markov model (HMM) file of the TCP domain (PF03634) was subsequently obtained from the Pfam database (Potter et al., 2018). The Simple HMM search tool in Tbtools was used to verify the presence of the conserved TCP domain in each candidate TCP protein. Finally, the integrity of the TCP domains will be assessed using the NCBI-CDD (https://www.ncbi.nlm.nih.gov/Structure/cdd/wrpsb.cgi). The members of the TCP family in *D. nobile* were determined after eliminating duplicate candidates (Marchler-Bauer et al., 2017). The online tools ProtParam in ExPASy (http://web.expasy.org/protparam/) and the Plant-mPloc (http://www.csbio.sjtu.edu.cn/bioinf/Cell-Ploc-2/) were employed to estimate the physicochemical properties and subcellular localization of TCP proteins in *D. nobile*, respectively (Chou and Shen, 2010; Artimo et al., 2012).

### 2.4 Chromosomal localization and collinearity analysis of TCPs

The chromosomal location and visualization of all TCP members of *D. nobile* were used by TBtools suite (Chen et al., 2020).The duplicate events of TCP genes were investigated and displayed on the *D. nobile* physical map via Multiple collinear scanning toolkits with the default parameters. In addition, the collinearity relationships between orthologous *TCP* genes of *D. nobile* and other species, the homo-linear analysis maps were constructed using Dual Synteny Plotter (Liu et al., 2019).

### 2.5 Conserved domains, conserved motif, and gene structure analysis of TCPs

The multiple protein sequences of identified TCP in *D. nobile* were aligned using MEGA X, and the alignment results were edited with Jalview (http://www.jalview.org/) (Clamp et al., 2004). The conserved regions of Class I and Class II TCP were submitted to the WebLogo to obtain protein sequence logos (Crooks et al., 2004). The TCP protein sequences were submitted to the MEME website (https://meme-suite.org/meme/tools/meme) to analyze conserved motifs. The analysis and visualization of the *DnTCP* gene structure of coding DNA sequences (CDS) were displayed in the gene structure view of TBtools using genome sequence and annotation information.

### 2.6 Phylogenetic relationship and miRNA−binding site recognition analysis of TCPs

To analyze the phylogenetic relationships between *TCP* genes in *D. nobile* and other species, we used the protein sequences of TCP from *D. nobile*, other orchid plants (*D. catenatum*, *P. equestris*, and *C. goeringii*), *Arabidopsis*, and rice. We constructed a phylogenetic tree using the neighbor-joining method (NJ) with MEGA X; the bootstrap test of phylogeny with 1000 replications. The online website Evolview (http://www.evolgenius.info/evolview) was used to enhance the visual appearance of the evolutionary tree. Potential miRNA319 and miRNA159 targeting *TCP* genes in *D. nobile* were predicted by the online software psRNATarget (http://www.zhaolab.org/psRNATarget/) with default parameters (Dai et al., 2018).

### 2.7 Cis-acting element and codon bias analysis of TCPs

The 2,000-base pair promoter sequences upstream of the start codon for all *TCP* genes were obtained from the genome database of *D. nobile*. The cis-elements and binding sites of these genes were determined using PlantCARE (http://bioinformatics.psb.ugent.be/webtools/plantcare/html/). and PlantPAN 4.0 (http://plantpan.itps.ncku.edu.tw/plantpan4/promoter_analysis.php). Meanwhile, the essential codon metrics such as effective number of codons (ENc), codon adaptation index/bias index (CAI/CBI), and frequency of optimal codons (Fop) were determined using the CodonW1.4.2 program (Wang et al., 2022; Fu and Liu, 2023). A heatmap clustering the relative synonymous codon usage (RSCU) of the TCP gene family in *D. nobile* was generated by using TBtools.

### 2.8 Protein interaction network and the GO annotation of TCPs

The TCP protein sequences of *D. nobile* were aligned with AtTCP proteins in *A. thaliana*, and the interactions of DnTCP proteins were predicted using the STRING database. Then, the interaction network was visualized by Cytoscape v3.7.1 (Shannon et al., 2003; Liu D. H. et al., 2022). Gene ontology enrichment analysis was performed, and the results were visualized using the OmicShare tool (https://www.omicshare.com/tools/).

### 2.9 RNA extraction and qRT-PCR

The perianth samples at four different developmental stages and from different organs of *D. nobile*, as mentioned in section 2.1, were collected for total RNA extraction. The RNA Simple Plant Kit (Huayueyang Biotechnology, China) was used for the extraction process. The cDNA synthesis and quantitative real-time PCR (qRT-PCR) were performed using the EasyScript^®^ One-Step gDNA Removal and cDNA Synthesis SuperMix (TransGen Biotech, China) and 2X TB Green™ Premix Ex Taq™ (TaKaRa, Japan), respectively. Relative gene expression was calculated using the 2^-ΔΔCT^ method (Livak and Schmittgen, 2001). We used 18S rRNA in *D. nobile* as a normalization control. Experiments were performed in triplicate. The primers used in qRT-PCR are listed in **Supplementary Table 2**. Statistical analyses were performed using the one-way ANOVA test in GraphPad Prism 9.0.

### 2.10 Subcellular localization

The CDS of the *DnTCP* genes, which did not contain stop codons, were cloned and inserted into the PHG vector to create the 35S:: DnTCPs-GFP recombinant vector (Han et al., 2022). Then, the fusion plasmids and the empty vector were transformed into GV3101 and subsequently infiltrated into tobacco leaves. The different fluorescence signals were observed using laser confocal microscopy (Zeiss LSM 880 Meta, Jena, Germany) after 48 hours. The primers and restriction sites are listed in **Supplementary Table 2**.

### 2.11 Yeast two-hybrid assay

To investigate the interactions between the Class I TCP and E class MADS-box proteins, we used the GAL4 two-hybrid system for the yeast two-hybrid assay (Y2H). The CDS of DnTCP9, DnTCP18, and DnSEP3-like were cloned into either the pGAD-T7 or pGBK-T7 vector. All recombinant vectors were co-transformed into AH109. The transformed yeast cells were grown on the SD/-Trp-Leu medium to select transformed positive clones at 29°C for 72 hours, and then the SD/-Trp-Leu-His-Ade (+X-α-gal) medium was used to confirm the positive interactions.

## 3 Results

### 3.1 Identification and traits of TCPs in *D.nobile*

In total, 23 TCP proteins were identified based on the *D. nobile* genome and named DnTCP1-23, and the complete protein sequences for DnTCP are listed in **Supplementary Table 3**. The 23 TCP proteins had an average length of 345 amino acids (range 211–786 aa). The average molecular weight (MW) was 37,488.93 kDa (range 22,320.27–88,497.66 kDa). The average isoelectric point (pI) was 7.91 (range 5.88–9.74), indicating a relatively high level of acidity. The aliphatic index of all DnTCP proteins was below 100, and their grand average of hydropathicity (GRAVY) was negative, indicating that they were highly hydrophilic. All TCP proteins were unstable, with instability coefficients exceeding 40.00. The majority of DnTCP proteins were predicted to be located in the nucleus. In addition to being localized in the nucleus, DnTCP2 may also be present in cell membranes, whereas DnTCP6 and DnTCP10 may also be found in chloroplasts (**Supplementary Table 3**).

### 3.2 Chromosome localization, gene duplication, and collinearity analysis

**Figure 1:**
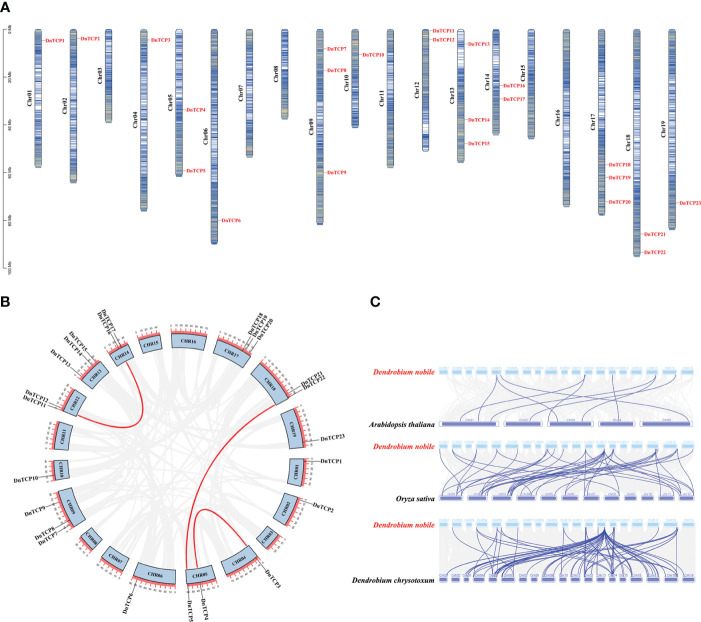
Chromosome localization, gene duplication, and collinearity analysis of *DnTCP* genes. **(A)** The figure shows the distribution of *DnTCP* genes from *D. nobile* across 19 chromosomes. The scale on the left indicates the chromosome length. The scale is in megabases, Mb. **(B)** Genomic locations and segmental duplication of the *DnTCP* genes. The red lines link paralogous *TCP* genes. **(C)** Collinearity of the *TCP* genes between *D. nobile* and *A thaliana*, *O. sativa*, and *D. chrysotoxum*. The blue lines indicate the *TCP* gene pairs.

Genomic analysis of *D. nobile* indicated that the species had 19 chromosomes, and 23 *DnTCP* genes were present on 13 chromosomes in an uneven pattern. Notably, the *DnTCP* genes were absent from chromosomes 03, 07, 08, 11, 15, and 16 (**Figure 1A**). Chromosomes 09, 13, and 17 contained the most genes, with each containing three *DnTCP* genes. Although chromosomes 01, 02, 04, 06, 10, and 19 each contain only one *DnTCP* gene, these genes were located near the poles of the chromosomes.

There are three pairs of segmentally duplicated genes in the TCP gene family of *D. nobile*, which are distributed on chromosomes 04, 05, 12, 14, and 18 (**Figure 1B**). Among these, *DnTCP3* and *DnTCP4*, *DnTCP5* and *DnTCP22*, as well as *DnTCP12* and *DnTCP16* are paralogous genes. The genes involved in segmental duplication events belonged to the CIN subclade. No tandem duplications of the *DnTCP* genes were found. These results suggest that segmental duplication is the predominant duplication event for *DnTCP* genes and is the primary driver of the expansion of the TCP gene family of *D. nobile*. This conclusion is consistent with previous studies on *Populus euphratica* (Ma et al., 2016), *Citrus sinensis* (Liu D. H. et al., 2022), and Ma Bamboo (Jin et al., 2022).

Collinearity analyses of the TCPs between *D. nobile* and other plant species were conducted to investigate the evolutionary relationships (**Figure 1C**). More collinear gene pairs were observed between the genomes of *D. nobile* and rice or *D. chrysotoxum* than between those of *D. nobile* and *A. thaliana*. Nine collinear gene pairs were found in the genomes of *D. nobile* and *A. thaliana*. However, large-scale orthologous TCP genes were found between the genomes of *D. nobile* and *D. chrysotoxum* (**Figure 1C**). These genes display not only synteny but also collinearity, which is a specific type of synteny where the genes are conserved in the same order (Tang et al., 2008). These results suggest that *TCP* genes may have a conserved evolutionary history in monocots and that those of *D. nobile* and *D. chrysotoxum* are highly similar.

### 3.3 DnTCPs conserved domain and motif, and gene structure

**Figure 2:**
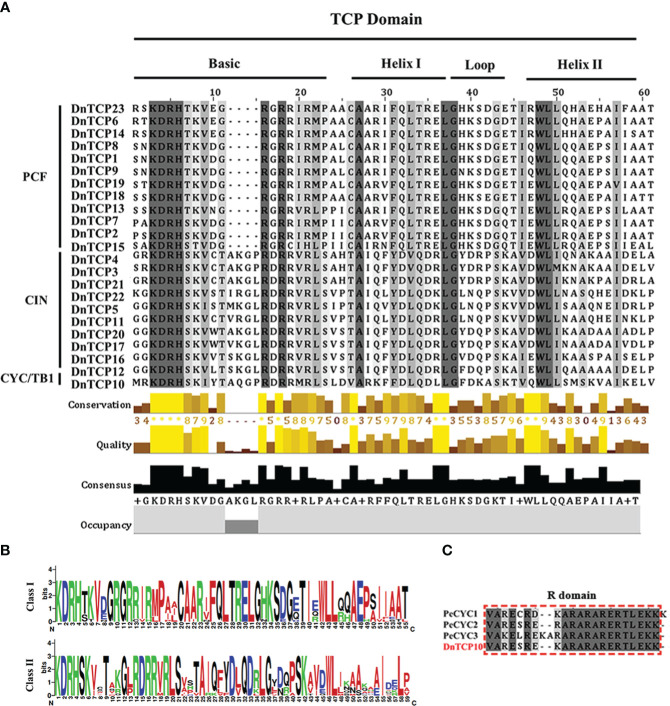
Multiple sequence alignment, the motifs, and the gene structures of DnTCPs. **(A)** The sequence alignment of TCP domains from *D. nobile*. The conserved regions (basic helix–loop–helix) the structure has been marked. **(B)** The sequence logo of TCP conserved regions. **(C)** Multiple sequence alignment of an R domain in TCP members. The conserved amino acids are in black.

**Figure 3:**
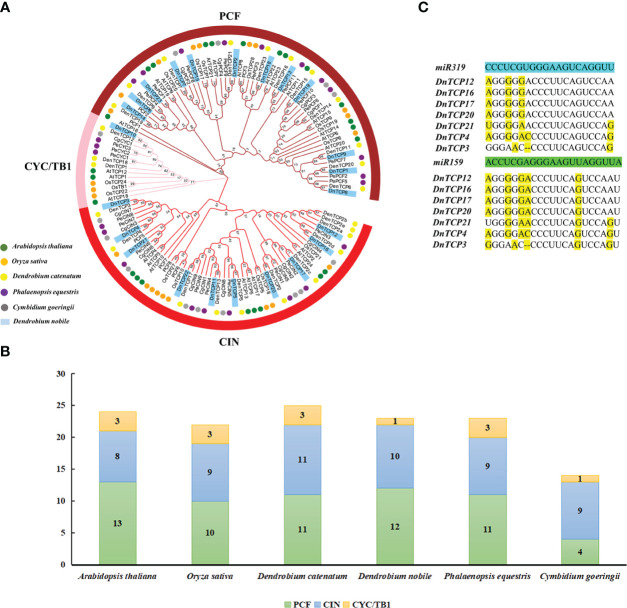
The phylogenetic analysis and miRNAs-binding site recognition of *DnTCP* genes. **(A)** The phylogenetic analysis of TCPs and among *D. nobile* (blue block), *A. thaliana* (green circle), *O. sativa* (orange circle), *C. goeringii* (gray circle), *P. equestris* (violet circle), and *D.catenatum* (yellow circle). The unrooted phylogenetic tree was constructed using the neighbor-joining (NJ) method implemented in the MEGA X software with 1000 bootstrap replicates. The brownish-red, pink, and red ring indicate PCF, CYC/TB1, and CIN subclade, respectively. **(B)** TCP family members and distribution pattern of *D. nobile*, *Arabidopsis*, *O. sativa*, *C. goeringii*, *P. equestris*, and *D.catenatum*. **(C)** Analysis of the putative target sites for miR319 and miR159 in DnTCP mRNAs. Mismatches were represented by yellow.

All sequences contained the TCP domain, which was confirmed by aligning them with 23 DnTCP protein sequences (**Figure 2A**). The results showed that DnTCP proteins could be divided into two classes, Class I (PCF) and Class II (CIN and CYC/TB1), as observed in all species studied thus far. According to this division, 12, 10, and 1 DnTCP members were clustered into PCF, CIN, and CYC/TB1, respectively. The phylogenetic analysis also supported this result (**Figure 3**). In the BASIC region, the amino acid residues Arg (R), His (H), Asp (D), and Lys (K) were completely conserved in the sequences of all members. However, as previously reported, the PCF clade had a four-amino acid deletion compared to Class II (**Figures 2A, B**). In addition, Class I and Class II exhibited internally conserved but distinct sequences in the loop regions (**Figures 2A, B**). The literature showed that *Phalaenopsis* has three CYC/TB1 genes (*PeCYC1*, *PeCYC2*, and *PeCYC3*) and the CIN gene *PeCIN3*, all of which contain an R-domain (Lin et al., 2016). However, in *D. nobile*, only one CYC/TB1 gene, *DnTCP10*, encodes a protein containing an R-domain (**Figure 2C**).

**Figure 4:**
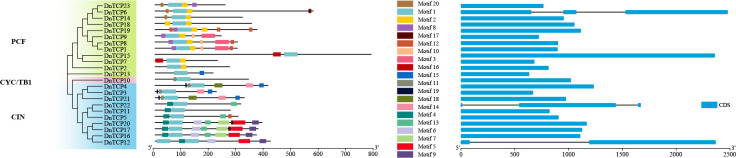
Conserved motif and gene structure of the *DnTCP* genes. Phylogenetic tree of 23 DnTCP proteins in the left; The conserved motifs of the *DnTCP* genes in the middle and each colored box represents a motif of the protein; exon–intron structures of DnTCP proteins in the right. Blue squares indicate CDS and black lines indicate introns.

Motif 1 was the most conserved in all DnTCP protein sequences (**Figure 4**). Motifs 2, 3, 8, 10, 16, and 20 were present only in the PCF clade. Motif 2 was present in all the PCF members, and DnTCP1 shared the same motif as DnTCP8 (**Figure 4**). The CIN clade members mainly consisted of motifs 4, 5, 6, 9, 13. DnTCP17 consisted with the motif of DnTCP20. DnTCP10 (CYC/TB1) comprised motif 1 and motif 11. In addition, most *DnTCP* genes contained only one exon and no introns in the coding regions (20/23, 86.96%), except for *DnTCP6*, *DnTCP12*, and *DnTCP22*. Among these, *DnTCP6* and *DnTCP22* both had three exons and two introns. However, *DnTCP12* had two exons and one longest intron (**Figure 4**).

### 3.4 The phylogenetic analysis and miRNA319-/159−binding site recognition of *DnTCP* genes

To analyze the evolutionary relationships of the TCP gene family in *D. nobile*, we constructed a phylogenetic tree. In total, 131 TCP protein sequences were identified in the rootless phylogenetic tree, including 24, 22, 14, 23, 25, and 23 TCP proteins from *A. thaliana*, *O. sativ*a, *C. goeringii*, *P. equestris*, *D. catenatum*, and *D. nobile*, respectively (**Figure 3A**). The homology of TCPs between monocots (Os, Cg, Pe, Dc, and Dn) and dicots (At) was low, and no AtTCP16 orthologs were identified in the five aforementioned monocot species (**Figure 3A**). This finding suggests a lineage-specific gene loss in *D. nobile* and implies that most *TCP* genes evolved before the divergence of dicotyledons and monocotyledons 140–150 million years ago (Chaw et al., 2004).

In *D. nobile*, the PCF clade contained the highest number of *TCP* genes, followed by the CIN clade, and the CYC/TB1 clade contained the fewest. The distribution pattern of the TCP gene family in this species is similar to that in *P. equestris* but different from that of *C. goeringii* (**Figure 3B**). Specifically, the CIN clade had the largest number of genes in *C. goeringii* (nine genes), and four genes were found in PCF, one-third of the number of PCF members in *D. nobile* (**Figure 3B**). This finding indicates that the CIN clade was more conserved than the PCF clade during orchid evolution.

Recently, several studies have described the function of miRNAs in some orchids, including *Phalaenopsis* (An et al., 2011), *Cymbidium* (Yang et al., 2017), and *Arundina graminifolia* (Ahmad et al., 2023), but not in *D. nobile*. Seven *DnTCP* genes (*DnTCP3*, *DnTCP4*, *DnTCP21*, *DnTCP12*, *DnTCP16*, *DnTCP17*, and *DnTCP20*) in the CIN clade contained putative binding sites for miR319 and miR159 (**Figure 3C**; **Supplementary Table 4**). In other plants, putative binding sites for miRNA319 and miRNA159 were also exclusively found in *CIN*-like genes (Lan and Qin, 2020; Ahmad et al., 2023). Our results indicate that miR319- and miR159-binding site sequences have been conserved throughout plant evolution.

### 3.5 Cis-acting regulatory elements and binding sites analysis of *DnTCP* genes

**Figure 5:**
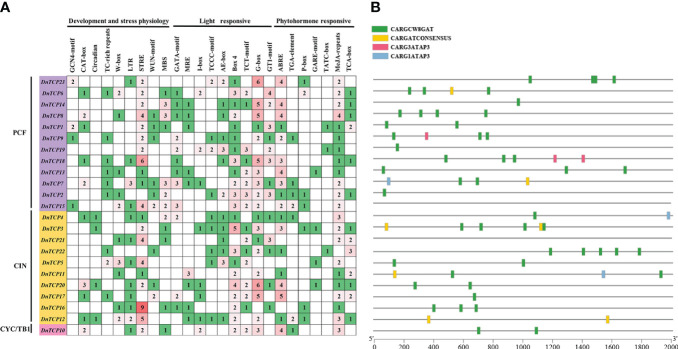
Cis-acting regulatory elements and MADS-box binding sites analysis of *DnTCP* genes. **(A)** Number of each cis-acting element in the promoter region of *DnTCP* genes. Based on the functional annotation, the cis-acting elements were classified into three main types: plant development and stress physiology, light-responsive, and phytohormone-responsive. **(B)** MADS-box binding sites (CArG-boxes) within the putative promoters of *DnTCP* genes of *D. nobile*. The different colors of rectangle represent indicate the different CArG-box variants, respectively.

Cis-acting regulatory elements (CAREs) control the precise initiation and transcription efficiency of gene transcription by binding to transcription factors (Yang et al., 2022). This study extracted the upstream regions 2,000 bp of *D. nobile TCP* genes to identify putative CAREs and investigate their functions. A total of 519 CAREs were identified in *D. nobile* (**Figure 5A**). Based on functional annotation, CAREs can be mainly categorized into three main types: plant development and stress physiology (155, 29.87%), light-responsive (232, 44.70%), and phytohormone-responsive (132, 25.43%).

CAREs functions include developmental elements such as endosperm expression, meristem expression, and circadian control, as well as stress responsiveness to factors such as salt, drought, and low-temperature stress. Interestingly, only four Class I *TCP* genes, *DnTCP1*, *DnTCP9*, *DnTCP15*, and *DnTCP23*, contained the GCN4 motif, which links to endosperm expression, indicating that reproductive development is closely related to the Class I *TCP* genes. Notably, *DnTCP18* and *TCP16* had six and nine stress response elements (STREs), suggesting that these two genes play essential roles in saline-alkali resistance.

All the DnTCPs had at least four additional CAREs associated with light responses. The most photoresponsive element was the G-box (59, 11.37%), followed by Box 4 (46, 8.86%) (**Figure 5A**). Box 4 was present in all *DnTCP* genes except for *DnTCP21*. Light responsiveness was the most prevalent functional element in *DnTCP* genes, indicating that light plays a crucial role in modulating TCP function during plant growth and development (Liu D. K. et al., 2022). In addition, various elements responding to abscisic acid (ABA), auxin, gibberellin (GA), methyl jasmonate (MeJA), and salicylic acid (SA) were also widely distributed in different *DnTCP* genes (**Figure 5A**). Interestingly, all *DnTCP* genes have at least one MeJA response element, except for *DnTCP22*, which possesses the most elements responsive to salicylic acid. These results suggest that the CAREs in *DnTCP* genes may have broad involvement in hormonal regulation while also being specific.

We found possible MADS-box binding sites (CArG boxes and its variants) existing in the putative promoter of *DnTCP* genes, which have the core consensus sequence CC(A/T)6GG (Folter and Angenent, 2006). **Figure 5B** shows that most *DnTCP* genes have CArG boxes, except for *DnTCP15* and *DnTCP21*. Among these genes, *DnTCP3* has the highest number of CArG boxes, a total of six, while *DnTCP2*, *DnTCP14*, and *DnTCP17* have only one each. *DnTCP7* and *DnTCP11* have the most CArG box types. Most *DnTCP* genes have CARGCW8GAT, except for *DnTCP12*, which has two CARGATCONSENSUS sites, possibly associated with flowering time (Hepworth et al., 2002). Interestingly, *DnTCP9* and *DnTCP18* are the only two *DnTCP* genes that have CARG3ATAP3 binding sites. This implies that they may play a specific role as downstream genes of certain MADS-box transcription factors in perianth development.

### 3.6 Codon usage bias and relative synonymous codon usage of *DnTCP* genes

The detailed results of codon-related parameters in the TCP gene family of *D. nobile* are listed in **Supplementary Table 5**. Among these, both the mean values of GC content (0.536) and GC of silent 3rd codon posit (GC3s) (0.517) are over 0.500, indicating that GC is used more frequently than AU in the codons. The average value of CAI was 0.70 (range 0.64–0.74), indicating that *DnTCP* genes exhibit significant bias toward codon selection. The average value of Fop was 0.454 (range 0.331–0.532). The average value of CBI was 0.050 (range -0.174–0.175). The average value of ENc was 52.92 (range 42.33–57.46). These results indicate that family members exhibit significant variation from each other, have comparatively moderate levels of expression, and have a low preference for specific codons when encoding amino acids.

**Figure 6:**
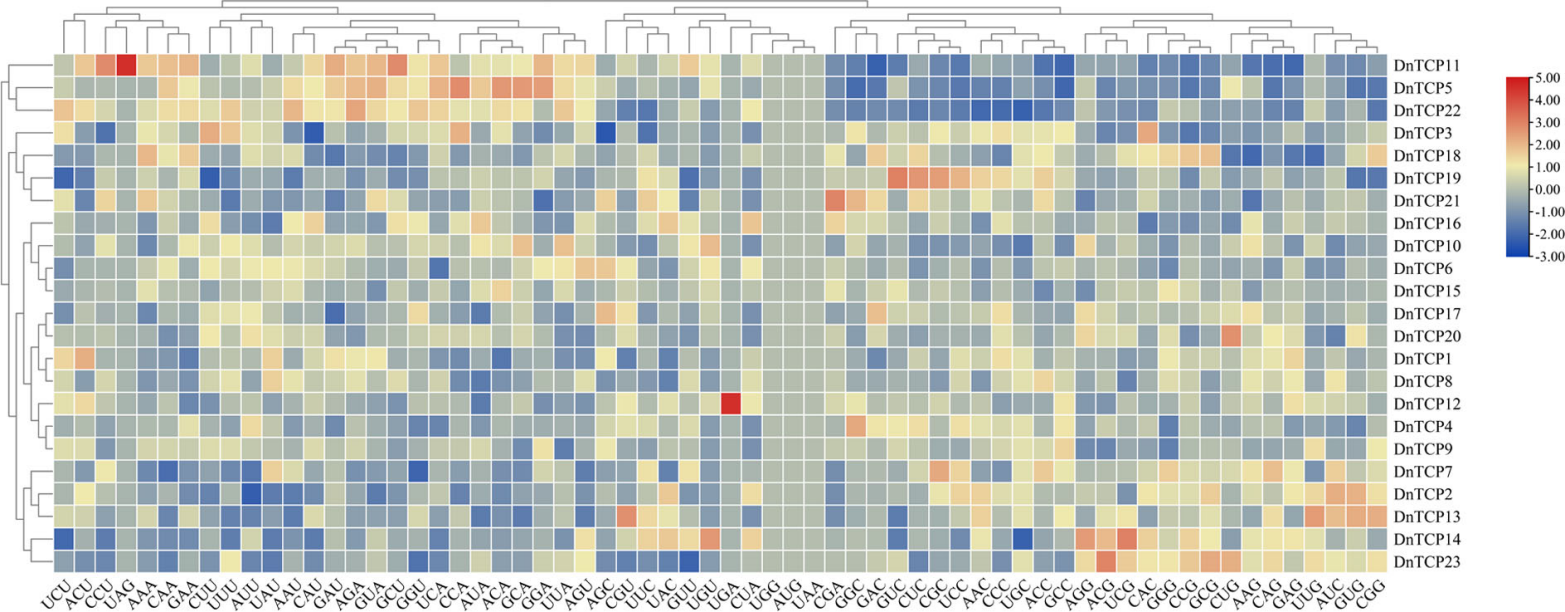
Heat map clustering of codons RSCU of *DnTCP* genes.

The RSCU of codons ranged from 0.39–1.71, excluding the termination and initiation codons. Among them, the RSCU value of CUC is the largest, which is 1.71, revealing that *D. nobile* TCP gene family members strongly prefer this codon (**Supplementary Table 6**). There are 28 high-use codons (RSCU>1), 11 of which end in C, 10 of which end in U, 5 of which end in G, and 2 of which end in A. This result implies that the preferences for high-use codons terminate with C, However, low-use codons terminate with A, which is significantly distinct from the results of the NAC gene family in *D.nobile* (Fu and Liu, 2023). The heatmap clustering of codons RSCU of *DnTCP* genes revealed that members exhibit similar codon usage biases in same branch (**Figure 6**).

### 3.7 Gene ontology classification and protein-protein interaction analysis of the DnTCPs

**Figure 7:**
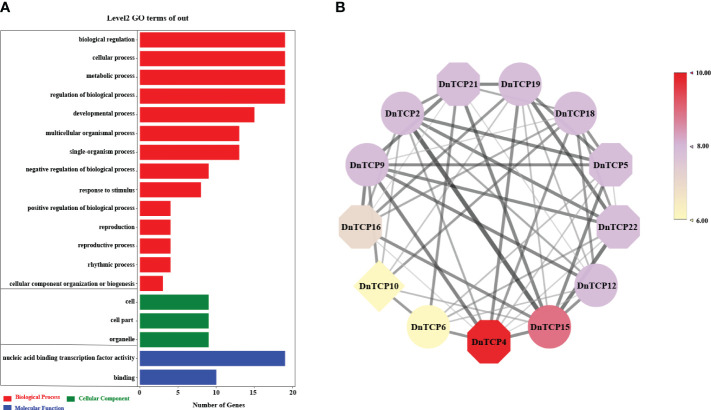
Gene ontology (GO) annotation and prediction of interaction network among DnTCPs. **(A)** GO annotations of DnTCPs. The red, green, and blue bars represent GO terms of biological process, cellular component, and molecular function, respectively. **(B)** Analysis of interaction network among DnTCPs. The circle, octagon, and diamond represent PCF, CIN, and CYC/TB1 subclade, respectively. Different color blocks represent the number of interaction proteins. The black line represents the combined score of two interaction proteins, and a higher score corresponds to a thicker line.

Gene ontology annotation was mainly divided into biological processes, cellular components, and molecular functions (**Figure 7A**). The majority of genes were enriched in biological processes. 19 *DnTCP* genes were enriched in biological process (GO: 0050789), biological regulation (GO: 0065007), metabolic process (GO: 0008152) and cellular process (GO: 0009987). In addition, genes were also enriched in the regulation of developmental process, rhythmic process, reproduction, single-organism process and so on. In cellular components, 9 *DnTCP* genes were enriched in organelles (GO:0043226), cell (GO: 0005623) and cell parts (GO: 0044464). At the same time, in terms of molecular function, 19 *DnTCP* genes were enriched in nucleic acid binding transcription factor activity, while 10 *DnTCP* genes were enriched in binding (**Figure 7A**).

Furthermore, the interaction relationships among the 23 DnTCP proteins were complex, and 13 members interacted with each other (**Figure 7B**). Among these, DnTCP4 interacted with ten members; DnTCP15 interacted with nine members; DnTCP2, DnTCP3, DnTCP5, DnTCP9, DnTCP11, DnTCP12, DnTCP18, and DnTCP19 interacted with eight members; DnTCP16 interacted with seven members; DnTCP6 and DnTCP10 interacted with six members. Moreover, the highest interaction score was found between DnTCP2 and DnTCP15 (**Supplementary Table 7**).

### 3.8 Spatio−temporal expression analysis of *DnTCP* genes

**Figure 8:**
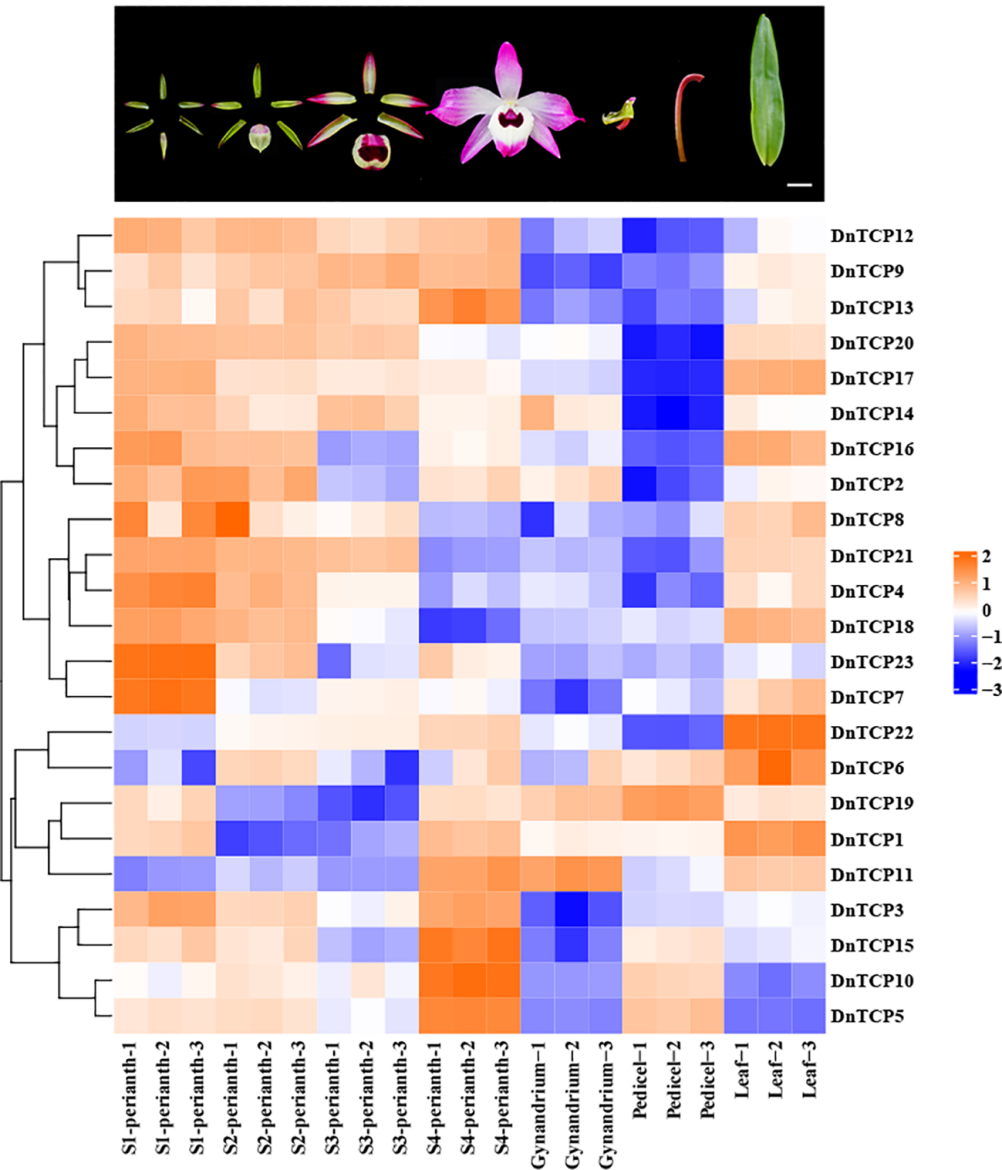
Heat map of the expression patterns of 23 *DnTCP* genes. The expression profile data by qRT-PCR. Expression values are log2-transformed. The expression levels are represented by the color bar.

**Figure 9:**
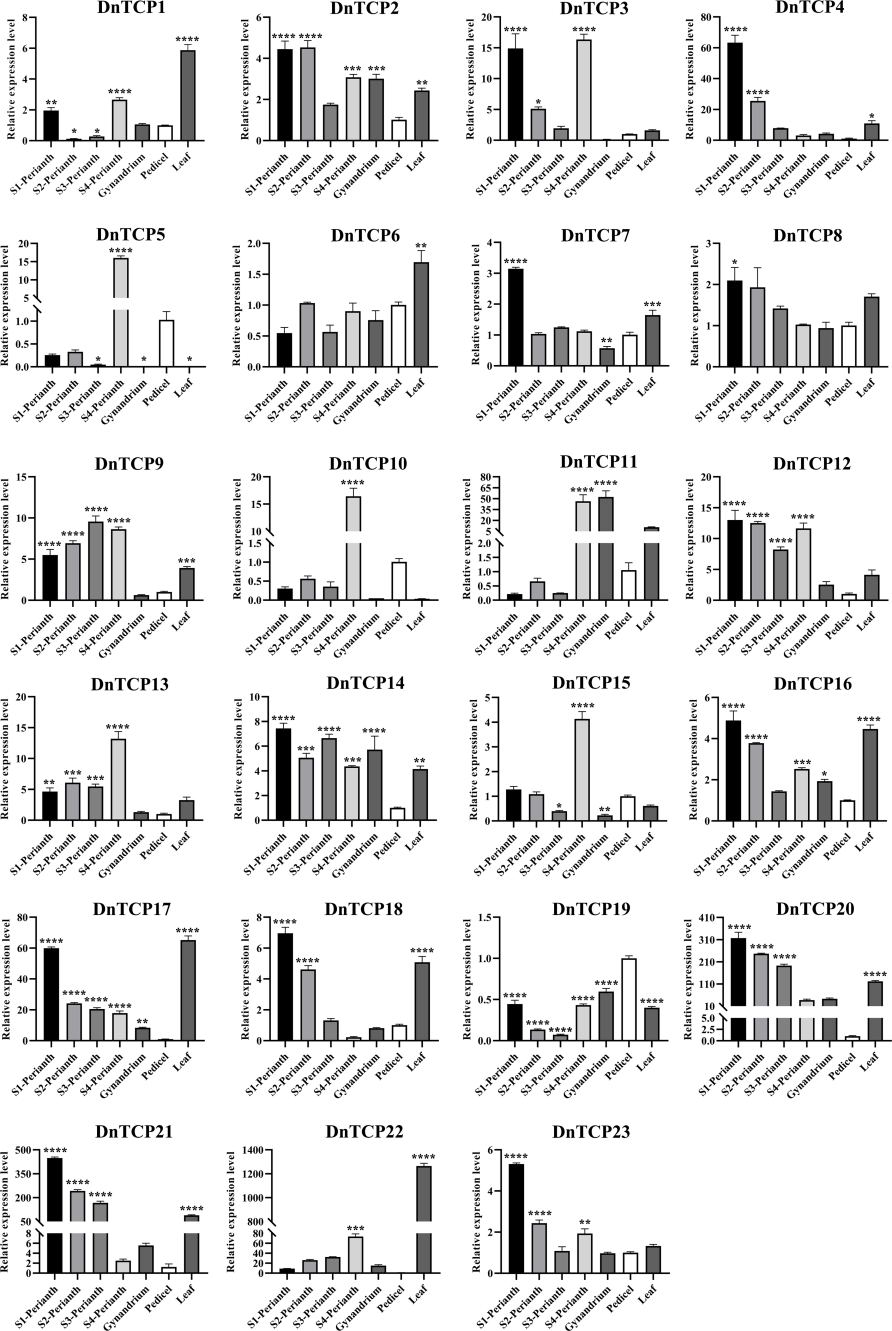
Spatio−temporal expression analysis of *DnTCP* genes at different perianth developmental periods or different organs of *D. nobile.* Relative values of gene expression were normalized to the expression of *18S rRNA*. Statistically significant differences were conducted using the one-way ANOVA test and indicated with *(P<0.05), **(P<0.01), ***(P<0.001), and ****(P<0.001). Error bars mean SD values.

Gene expression patterns are correlated with gene functions. As shown in the **Figures 8** and **9**, there were six *DnTCP* genes (*DnTCP4*, *DnTCP8*, *DnTCP17*, *DnTCP18*, *DnTCP20*, and *DnTCP21*) showed similar expression patterns at different stages of perianth development and exhibited the highest expression level in S1 and the lowest expression level in S4. This finding suggests that these genes are associated with early floral meristem development and flowering. However, the expression levels of *DnTCP5*, *DnTCP10*, *DnTCP11*, *DnTCP13*, *DnTCP15*, and *DnTCP22* at S4 were significantly higher than those at the preceding three stages (**Figures 8**, **9**). These genes might play a role in maintaining perianth expansion during the late stages of perianth development by promoting cell division. In addition, *DnTCP9* and *DnTCP12* were continuously and steadily expressed during the four stages of perianth development, and there were no significant differences in their expression levels among the stages (**Figures 8**, **9**).

In summary, the expression levels of CIN-like genes were higher than those of the PCF clade at different stages of perianth development (**Figures 8**, **9**). In particular, *DnTCP17*, *DnTCP20*, *DnTCP21*, and *DnTCP22* were highly expressed in both perianth and leaves, suggesting that these genes may play a role in regulating perianth and leaf development. Notably, because *DnTCP17* and *DnTCP20* have similar motif distributions or gene structures, and their expression patterns are clustered on the same branch (**Figures 4**, **9**), they may be involved in growth and development in a functionally redundant manner. *DnTCP5* and *DnTCP10* were not expressed in the leaves or gynostemium. However, *DnTCP11* was significantly expressed in the gynostemium (**Figures 8**, **9**), suggesting that *DnTCP11* plays a role in regulating plant reproduction.

### 3.9 Subcellular localization of DnTCP9 and DnTCP18

**Figure 10:**
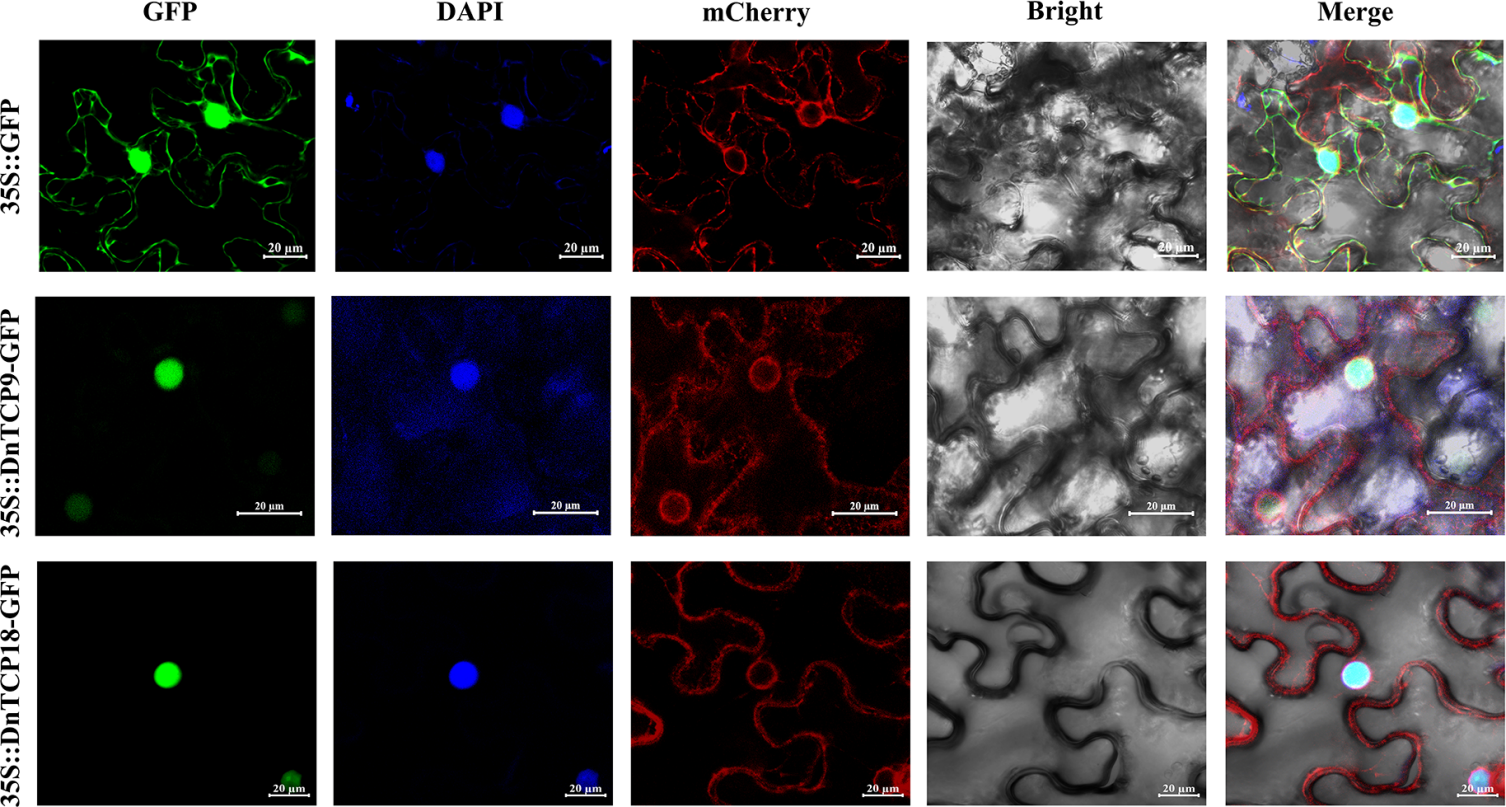
Subcellular localization analysis of DnTCP9 and DnTCP18 in *Nicotiana benthamiana*. Localization results of 35S:: GFP empty plasmid as the control. Co-localization of 35S:: DnTCP9-GFP or DnTCP18-GFP with nucleus marker DAPI and endoplasmic reticulum marker ER-mCherry. Scale bar = 20 µm.

In various plant species, members of the TCP gene family act as transcription factors that regulate plant growth and development (Ma et al., 2016; Lan and Qin, 2020; Zhang et al., 2020; Viola et al., 2023). Since transcription factors regulate the transcription of target genes within the nucleus, DnTCPs are likely to be localized in the nucleus. Some studies have analyzed the subcellular localization of Class I TCPs (Jin et al., 2022; Yang et al., 2022; Ren et al., 2023). In this study, Class I TCPs, especially DnTCP9 and DnTCP18, were selected to investigate their subcellular localization, which could have unique roles in perianth development via binding to specific MADS-box. As shown in **Figure 10**, 35S:: DnTCP9-GFP and 35S:: DnTCP18-GFP were localized to the nucleus according to the fluorescence signals, consistent with the results consistent with the prior hypothesis (**Supplementary Table 3**). In addition, DnTCP9 showed weak fluorescence signals in the endoplasmic reticulum, indicating many unique situations of subcellular localization of transcription factors (Yang et al., 2022).

### 3.10 Interaction detection between DnTCPs and between DnTCPs and SEP3

Functional studies of TCPs and their interaction factors are crucial for understanding their role in controlling specific plant growth and development. SEP3 (previously known as AGL9, E-class MADS-box) is positioned at the apex of the regulatory hierarchy of the MADS-box and plays a crucial role in flower development and the identity of floral organs (Chang et al., 2009; Wang et al., 2019). Therefore, we identified a sequence highly homologous to SEP3 in *D. nobile*. The cDNA of an E-class *MADS* gene (KAI0523144.1) was isolated from the genome. According to the result of the phylogenetic tree with the SEP3 protein sequences identified from other plants (**Supplementary Figure 1**), this gene belonged to the SEP3-clade, thus, we named this gene *DnSEP3-*like.

To determine whether the two Class I members, DnTCP9 and DnTCP18, interact with each other and whether they can also interact with DnSEP3-like, a protein associated with flower development, we conducted a Y2H experiment. After excluding the self-activation phenomenon of BD-DnTCP9 and BD-DnTCP18, we performed interaction detection by combining BD-DnTCP9 with AD-DnTCP18/DnSEP3-like, respectively. The same applies to BD-DnTCP18 with AD-DnTCP9/DnSEP3-like.

**Figure 11:**
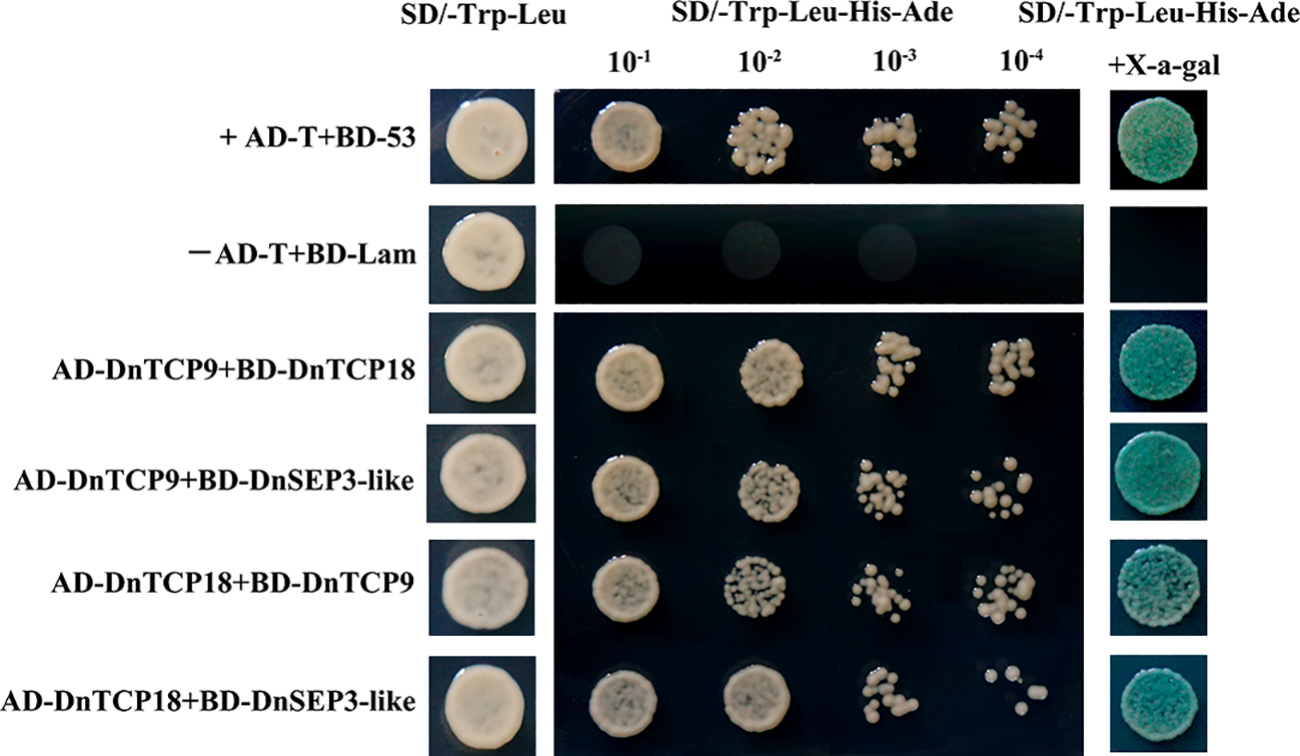
Yeast-two-hybrid analysis of protein–protein interactions among DnTCP proteins or DnTCP proteins and DnSEP3-like. AD-T/BD-53 and AD-T/BD-Lam are the positive and negative controls, respectively.

All combinations maintained well on the SD/-Trp-Leu solid medium, as shown in **Figure 11**, demonstrating successful co-transformation. Furthermore, all co-transformed yeast strains that was grown on the SD/-Trp-Leu-His-Ade selective medium also tinted the substrate blue in the X-a-gal medium, suggesting that DnTCP9 and DnTCP18 interact not only with each other but also with DnSEP3-like proteins.

## 4 Discussion

Here, we identified 23 *DnTCP* genes that were unevenly distributed on 19 chromosomes of *D. nobile* (**Figure 1A**). These genes included 12 members of the PCF clade, 10 members of the CIN clade, and 1 member of the CYC/TB1 clade. The TCP gene family of *D. nobile* typically exhibits conserved TCP domains and motifs (**Figures 2**, **4**). Genes with similar motif distributions or gene structures may have been evolutionarily conserved and perform the same functions. Consequently, *DnTCP1* and *DnTCP8* (PCF clade), and *DnTCP17* and *DnTCP20* (CIN clade), might contribute to the functional redundancy observed among members of the same subclade. Each subclade exhibited unique characteristics in its combination of conserved motifs. For example, motif 4 was present in almost all of Class II CIN proteins, but motifs 2, 3, 8, and 20 were exclusive to the PCF subclade. Additionally, DnTCP10 (CYC/TB1 clade) has an R-domain (**Figure 2C**), which may be related to protein-protein interactions and phosphorylation process (Dulhanty and Riordan, 1994; Cubas et al., 1999). However, its specific functions require further investigation.

The total number of TCP gene families and their distribution patterns are similar to those observed in *A. thaliana* (24), *O. sativa* (22), *P. equestris* (23), and *D. catenatum* (25) (**Figures 3A, B**). This result may be because the genome size of *D. nobile* is similar to that of *P. equestris* and *D. catenatum*, which are 1.19 Gb, 1.13 Gb, and 1.11 Gb, respectively (Cai et al., 2015; Zhang et al., 2016; Xu et al., 2022). Also, we found that the phylogenetic relationships of the *DnTCP* genes were close to those of *P. equestris* and *D. catenatum*. These genes almost clustered into the same branch as *DnTCP18*, *DenTCP23*, and *PePCF3* (**Figure 3A**). Although the genome of *D. nobile* is approximately 9.5 and 2.6 times larger than those of *Arabidopsis* and *O. sativa*, respectively, the number of genes in the genomes is analogous (Cai et al., 2015). This finding implies that the TCP gene family did not expand along the genome expansion of *D. nobile* during orchid evolution.

Conversely, the total number of TCP gene family members in *D. nobile* differs significantly from that in *C. goeringii* (14) (**Figure 3B**). This significant difference in members was also observed between *D. nobile* and *D. chrysotoxum*, as well as, between *D. nobile* and *D. huoshanense*, which was 1.5 and 2 times, respectively (Huang et al., 2023), and was mainly due to the number of *TCP* genes in the PCF or CYC/TB1 clade. These results indicate that *TCP* gene duplication and loss occurred during orchid evolution, while the CIN clade gene may be more conserved than the other clades. In addition, our analysis of codon use bias and relative synonymous codon usage offers a new perspective for analyzing evolutionary relationships in *Dendrobium* (**Figure 6**), and it can be utilized to increase gene expression efficiency in genetic transformation research (Wang et al., 2022).

*DnTCP* genes possess many cis-acting elements connected to light sensitivity, hormonal responses, and stress responses (**Figure 5A**). These TCPs are speculated to play a role in the growth and development of *D. nobile*, stress tolerance, and hormone signaling. This assumption is also supported by the GO prediction (**Figure 7A**). *Dendrobium* orchids have developed multiple photosynthetic pathways, as the CAM pathway has evolved at least eight times independently (Li et al., 2019; Xue et al., 2023). Thus, the promoters of *DnTCP* genes contain many cis-acting elements associated with light sensitivity. Additionally, the promoters of *DnTCP* genes contain many STREs and ABA response elements (ABREs), which correspond to the efficient utilization of water in CAM plants. Hormones are mediators of TCP-regulated growth and development. Liu et al. (2020) found that PeTCP10 in moso bamboo has the potential to positively regulate drought tolerance via an ABA-dependent signaling pathway and negatively affect lateral organ growth via the MeJA-mediated signaling pathway. In addition, heterologous overexpression of *CnTCP9* gene may affect plant traits such as petals and leaves via the GA signaling pathway (Yu et al., 2022). Some *Dendrobium* TCP transcription factors have been involved in plant hormone responses, including ABA, MeJA, and SA (Zhang et al., 2021b; Huang et al., 2023). In this study, many of the above hormone-responsive elements were found in different *DnTCP* genes, suggesting that they may be involved in signaling cascades that control hormonal pathways to regulate plant development. However, further research is needed to fully understand the intricate relationships between *TCP* genes, hormones, and plant growth and development in *D.nobile*.

miRNAs are fundamental regulators of plant growth and development. A subset of *CIN*-like genes in many plants analyzed to date are targets of the miR319 and miR319-TCPs regulatory systems, which are widely involved in the development of plant roots (Baulies et al., 2022), leaves (Koyama et al., 2017; Sun et al., 2022), flowers (Nag et al., 2009; Silva et al., 2019), and plant immunity (Lopez et al., 2015). Interestingly, we found that miR159 shares the same target genes as miR319. The genes belonged to the CIN clade (**Figure 3C**). The miRNA319 and miRNA159 families share significant similarities in their base sequences. MiRNA159 is cross-regulated with miRNA319 and targets several *MYB* genes that regulate flower development (Rubio-Somoza and Weigel, 2013; Hong and Jackson, 2015). Additionally, miRNA159-targeted *TCP* and *MYB* showed similar expression patterns during early flower development in *A. graminifolia* (Ahmad et al., 2023). As a result of relative expression analysis, we found that DnTCP3, DnTCP17, DnTCP20, and DnTCP21, which are co-targeted by miRNA319 and miRNA159, were highly expressed in S1 (**Figures 8**, **9**). Therefore, a hypothesis is that these DnTCPs play a role in early perianth development by acting alongside MYB in a complex regulatory network. Previous studies have shown that overexpression of miRNA319 leads to the development of larger leaves with crinkled surfaces (Lan and Qin, 2020). This phenomenon is due to the increased cell proliferation along the margins of the leaves, which is achieved by down-regulating the targeted CIN-like genes *AtTCP2*, *AtTCP3*, *AtTCP4*, *AtTCP10*, and *AtTCP24* (Palatnik et al., 2003). We found that DnTCP17 and DnTCP20 were highly expressed in leaves and shared homology with *AtTCP2* and *AtTCP24* (**Figures 8**, **9**). This finding suggests that leaf development in *D. nobile* is regulated by a redundant set of miRNA-regulated homologous *TCP* genes. Indeed, the *CIN*-like genes that are not targeted by miRNA319 show broad expression at different perianth stages and organs. Among these genes, the highest relative expression of *DnTCP22* was observed in leaves (**Figures 8**, **9**), and three salicylic acid-responsive elements were found in the promoter region (**Figure 5A**). In addition, *AtTCP13* and its two closely related homologs, *AtTCP5* and *AtTCP17*, orthologs of *DnTCP22*, are strongly transcribed in leaves and play a role in mediating leaf differentiation (Hur et al., 2019). These results suggest that DnTCP22 may be involved in leaf development and senescence (Buchanan-Wollaston et al., 2005).

Class I TCPs, such as AtTCP8, 20, and 22, promote cell differentiation or proliferation in *Arabidopsis*, and PePCF10 is also hypothesized to play a similar role in *Phalasenopsis* (Aguilar-Martínez and Sinha, 2013; Lin et al., 2016). However, *DnTCP19*, a homologous gene of *PePCF10*, showed low expression at different developmental stages of the perianth and in organs (**Figures 8**, **9**). It cannot be ruled out that gene function might be species-specific, or another gene from the Class I (PCF) clade might replace *DnTCP19*. For example, during perianth development, Class I gene, *DnTCP9*, showed successive expression patterns, while *DnTCP18* exhibited higher expression levels than other Class I genes at the early stages of perianth development (S1 and S2) (**Figures 8**, **9**). A homolog of *DnTCP9*, known as *AtTCP20*, may be involved in petal development by regulating cell expansion, division, and differentiation (Hervé et al., 2009; Danisman et al., 2012).In short, these two Class I TCPs may contribute to the unique functions in orchids, particularly in regulating perianth development as nuclear transcription factors (**Figure 10**).

The regulatory framework of CYC, which controls the formation of floral symmetry, has been extensively studied in core eudicots. Floral zygomorphy formation depends on the asymmetric dorsoventral expression of *CYC*-like genes (Hileman, 2014). In Phalaenopsis, there are three CYC/TB1 genes, but only *PeCYC1* and *PeCYC2* are differentially expressed in the dorsal and ventral petals. However, we found only a single copy of the CYC/TB1 clade, known as *DnTCP10*, which is consistent with studies conducted on other orchids such as *Orchis italica* and *Cattleya* (De Paolo et al., 2015; Madrigal et al., 2017). *DnTCP10* was highly expressed only at the later stages of perianth development after floral zygomorphy was established (**Figures 8**, **9**). It has been speculated that *DnTCP10* does not play a major role in floral zygomorphy but is involved in lateral bud development, similar to the function of CYC/TB1 genes in *Arabidopsis* (Aguilar-Martínez et al., 2007). Flower symmetry and petal identity of Delphinieae flowers originated from the rewiring of interactions between duplicated and diversified *TCP* and *MADS-box* genes, resulting in a nearly zygomorphic flower (Zhao et al., 2023). Similar results were found in Asteraceae and Papaveraceae (Zhao et al., 2018; Xiong et al., 2023). These results inspired us to explore the origins of floral symmetry and perianth diversity in *D. nobile*. Further experiments are needed to verify *TCP* and *MADS-box* gene expression patterns and gene functions in perianth organs, including sepals, petals, lips (specialized petals), and lateral organs, as well as the complex regulatory links between TCP and MADS-box.

TCPs can form dimers or even multimers interacting with several other proteins (Valsecchi et al., 2013). The Y2H results suggest that two Class I proteins, DnTCP9 and DnTCP18, interact with each other (**Figure 11**) as a result of protein-protein interactions using the String database (**Figure 7B**). This finding is supported by those of Danisman et al. (2012) in *Arabidopsis*, in which protein interactions between AtTCP20-AtTCP8 and AtTCP20-AtTCP22 were observed. In addition, TCPs may cooperate with the MADS-box and play key roles in the regulation of flowering, floral organ identity, and flower symmetry through protein interactions or promoter binding (Yuan et al., 2020; Zhao et al., 2020; Lan et al., 2023). However, these studies have primarily investigated the correlation between Class II TCP and MADS-box but not that for Class I TCPs. Therefore, we investigated the relationship between Class I TCP proteins and DnSEP3 (an E-class MADS-box) in *D. nobile*. We are the first to investigate this relationship and have thus filled a gap in previous orchid research. Our findings showed that DnSEP3 interacted with DnTCP9 and DnTCP18 (**Figure 11**). SEP-like acts as a binding agent for other MADS-box proteins (Hugouvieux and Zubieta, 2018). Moreover, it is also necessary for the identity and meristem development of the perianth organs in orchids such as *Dendrobium* (Yu and Goh, 2000), *Habenaria radiata* (Mitoma and Kanno, 2018), and *Cymbidium sinense* (Lin et al., 2023). A reasonable assumption is that the two Class I TCP proteins, DnTCP9 and DnTCP18, form dimers or heterodimers with DnSEP3; they might also form higher-order protein complexes with other types of MADS-box proteins (Xu et al., 2006), providing a flexible mechanism for the regulation of perianth development in *D. nobile*. Notably, Wen et al. (2019) found that ClCYC2 interacts with most ABCE-class MADS-box proteins to regulate capitulum development in *chrysanthemum*, except for ClSEP3. However, the TCP4 (CIN clade) transcription factor interacts directly with the AG-SEP3 complex, which determines the fate of the carpels in *A. thaliana* and inhibits their function, suggesting that the different clades of TCPs in various species may employ distinct strategies to interact with SEP3-like to control flower development (Lan et al., 2023).

Alternatively, MADS-specific binding sites (CArG boxes) are present in the majority of *DnTCP* putative promoters (**Figure 5B**), indicating that TCP and MADS-box may interact at the transcriptional level, with MADS-box acting as transcriptional repressors or activators of *DnTCP* genes (Lucibelli et al., 2021). Lin et al. (2023) found that the transcriptional activation levels of *AtTCP3* and *AtTCP20* were an upregulated expression in transgenic lines that overexpressed *CsSEP3* gene with curled leaf phenotypes. This result hints that the TCP gene can also act as a target gene of SEP3 in orchids, as has been observed in *Arabidopsis* and gerbera (Kaufmann et al., 2009; Zhao et al., 2020). TCPs were also capable of altering the expression domain of B-class *MADS-box* genes in the perianth, which play an important role in determining petal size and are essential for perianth organ identity in *Cysticapnos* (Zhao et al., 2018). However, the involvement of TCP and MADS-box transcription factor networks in flower development is complicated and often involves multiple regulatory mechanisms. For instance, in Delphinieae, two *CYC2-*like genes, *DeajCYC2b* and *DeajCYC2a*, are involved in petal development through different regulatory mechanisms. DeajCYC2b likely acts as a direct positive regulator of MADS-box, while *DeajCYC2a* acts as a downstream gene regulated by the MADS-box, forming a complex feedback loop with MADS-box and MYB (Zhao et al., 2023). Furthermore, several studies have indicated that MADS-box proteins form homodimers and/or heterodimers bind to CArG-box (Riechmann et al., 1996; Tilly et al., 1998; Pan et al., 2014). Thus, it would be interesting to find out whether the heterodimers formed by DnTCP9 or DnTCP18 with DnSEP3 can regulate growth and development by controlling the gene transcript level of other *DnTCP* genes.

## 5 Conclusions

In this comprehensive study, we found that 23 *DnTCP* genes were unevenly distributed on 19 chromosomes in *D. nobile*. These *DnTCP* genes were classified into Class I (PCF) and Class II (CIN and CYC/TB1 subclades) according to their phylogenetic and structural characteristics. DnTCPs are closely related to *D. catenatum* TCPs. DnTCPs may have unique roles due to differences in gene structure, motif composition, protein interaction traits, and expression patterns. DnTCP10 (CYC/TB1) may not regulate the bilateral perianth. Four CIN clade genes, DnTCP17, DnTCP20, DnTCP21, and DnTCP22, most likely control perianth and leaf development. Additionally, miR319 and -159 co-target *DnTCP17*, *DnTCP20*, and *DnTCP21* genes. As nuclear transcription factors, two Class I DnTCPs, DnTCP9 and DnTCP18, interact with each other or with DnSEP3-like protein. Moreover, their promoter sequences contain MADS-box binding sites, suggesting that TCP-MADS might employ intricate and flexible strategies in the regulation of perianth development and identification of perianth organs.

In brief, our study demonstrates a specific role for TCP genes and increases the understanding of their functions during perianth development in *D. nobile*. Based on the results of this study, an exploration of more important DnTCPs should be conducted to create models of their upstream and downstream regulatory workflows. This further research will facilitate comprehending the diverse perianth molecular networks in orchids.

## Data availability statement

The datasets presented in this study can be found in online repositories. The names of the repository/repositories and accession number(s) can be found below: https://www.ncbi.nlm.nih.gov/, PRJNA725550.

## Author contributions

XW: Data curation, Software, Validation, Visualization, Writing – original draft. MY: Data curation, Software, Visualization, Writing – review & editing, Formal analysis. BZ: Project administration, Resources, Supervision, Writing – review & editing. LZ: Resources, Supervision, Writing – review & editing. YW: Funding acquisition, Resources, Supervision, Writing – review & editing.
