# Supplementary material for: Genome-wide identification and characterization of TCP gene family in Dendrobium nobile and their role in perianth development
Source: Front Plant Sci. 2024 Feb 5;15:1352119. doi: 10.3389/fpls.2024.1352119 (PMC10875090; doi:10.3389/fpls.2024.1352119)
Supplement: Supplementary file 1 [file DataSheet_1.docx]

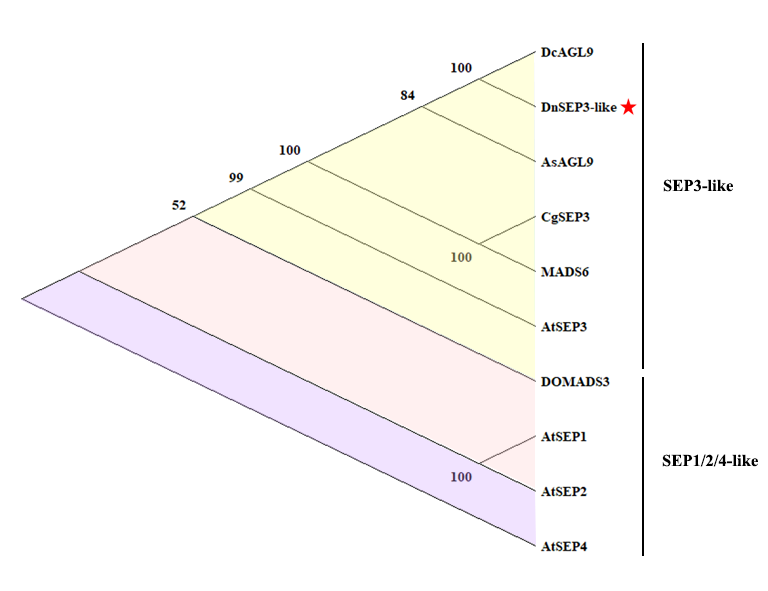


Figure S1. Classification and phylogenetic tree of SEP-like proteins of *Dendrobium nobile* (DnSEP3-like, KAI0523144.1), *D. catenatum* (DcAGL9, PKU79144.1), *Cymbidium goeringii* (CgSEP3, AHJ80843.1), *Apostasia shenzhenica* (AsAGL9, PKA62057.1), *D.hybrid* (DOMADS3, AAF13262.1), *Oncidium hybrid* (MADS6, ADJ67238.1), and *Arabidopsis thaliana* (AtSEP3, AT1G24260.2; AtSEP1, AT5G15800.2; AtSEP2, AT3G02310.1; AtSEP4, AT2G03710.1). The DnSEP3-like was marked with red stars.
